# Intracellular Ca^2+^-handling differs markedly between intact human muscle fibers and myotubes

**DOI:** 10.1186/s13395-015-0050-x

**Published:** 2015-08-20

**Authors:** Karl Olsson, Arthur J. Cheng, Seher Alam, Mamdoh Al-Ameri, Eric Rullman, Håkan Westerblad, Johanna T. Lanner, Joseph D. Bruton, Thomas Gustafsson

**Affiliations:** Department of Laboratory Medicine, Karolinska Institutet, Karolinska University Hospital Huddinge, Stockholm, 141 86 Sweden; Department of Physiology and Pharmacology, Karolinska Institutet, von Eulers väg 8, Stockholm, 171 77 Sweden; Department of Molecular Medicine and Surgery, Karolinska Institutet, Karolinska University Hospital Solna, Stockholm, 171 76 Sweden

**Keywords:** Human skeletal muscle, Ex vivo model, Intact human muscle fibers, Satellite cells, Myoblasts, Excitation-contraction coupling, Force production, Gene expression, Metabolic processes

## Abstract

**Background:**

In skeletal muscle, intracellular Ca^2+^ is an important regulator of contraction as well as gene expression and metabolic processes. Because of the difficulties to obtain intact human muscle fibers, human myotubes have been extensively employed for studies of Ca^2+^-dependent processes in human adult muscle. Despite this, it is unknown whether the Ca^2+^-handling properties of myotubes adequately represent those of adult muscle fibers.

**Methods:**

To enable a comparison of the Ca^2+^-handling properties of human muscle fibers and myotubes, we developed a model of dissected intact single muscle fibers obtained from human intercostal muscle biopsies. The intracellular Ca^2+^-handling of human muscle fibers was compared with that of myotubes generated by the differentiation of primary human myoblasts obtained from vastus lateralis muscle biopsies.

**Results:**

The intact single muscle fibers all demonstrated strictly regulated cytosolic free [Ca^2+^] ([Ca^2+^]_i_) transients and force production upon electrical stimulation. In contrast, despite a more mature Ca^2+^-handling in myotubes than in myoblasts, myotubes lacked fundamental aspects of adult Ca^2+^-handling and did not contract. These functional differences were explained by discrepancies in the quantity and localization of Ca^2+^-handling proteins, as well as ultrastructural differences between muscle fibers and myotubes.

**Conclusions:**

Intact single muscle fibers that display strictly regulated [Ca^2+^]_i_ transients and force production upon electrical stimulation can be obtained from human intercostal muscle biopsies. In contrast, human myotubes lack important aspects of adult Ca^2+^-handling and are thus an inappropriate model for human adult muscle when studying Ca^2+^-dependent processes, such as gene expression and metabolic processes.

**Electronic supplementary material:**

The online version of this article (doi:10.1186/s13395-015-0050-x) contains supplementary material, which is available to authorized users.

## Background

Satellite cells are resident stem cells of the skeletal muscle that can be isolated from human muscle biopsies, activated into proliferating myoblasts, and differentiated into multinuclear myotubes ex vivo [1, 2]. Myotubes have been credited with morphological, metabolic, and biochemical properties similar to those in adult skeletal muscle fibers [3, 4]. This in combination with the difficulty of obtaining intact viable muscle fibers from humans have rendered myotubes a widely used model to study aspects of cell signaling and metabolism in human skeletal muscle [3, 5–8].

In addition to their reported similarities to adult muscle fibers, myotubes also exhibit features that distinguish them from adult muscle. These include the expression of immature forms of muscle proteins [9], differences in abundance and distribution of glucose transporters [10, 11], and dominance of anaerobic glycolysis [11] as compared with adult muscle fibers. Furthermore, although human myotubes can obtain contractile ability under specific culture conditions, generated contractile forces are lower, fused tetani are attained at lower stimulation frequencies, and kinetic parameters are slower than those in adult muscle fibers [12–14]. This suggests an immature intracellular Ca^2+^-handling in myotubes, a notion supported by findings in previous studies investigating the Ca^2+^-handling properties of human myotubes [15–17]. To date, there is a lack of knowledge regarding the quantitative aspects of these differences since no direct comparison between human intact muscle fibers and myotubes has been previously performed.

Extensive evidence demonstrates the importance of intracellular Ca^2+^ for the regulation of a wide range of skeletal muscle processes, including muscle force generation, gene expression, and cellular metabolism [18–21]. Given the importance of cytosolic free [Ca^2+^] ([Ca^2+^]_i_) transient amplitude and duration [22] as well as frequency of [Ca^2+^]_i_ transients [21] for the downstream effects, differences in Ca^2+^-handling properties will have important implications when translating knowledge gained in myotubes to adult muscle fibers. Thus, we considered that a thorough characterization of the intracellular Ca^2+^-handling in human muscle fibers and myotubes was warranted in order to quantify the extent to which human myotubes differ in their Ca^2+^-handling properties compared to adult muscle fibers.

In the present study, we hypothesized that human muscle fibers and myotubes would display major functional differences in intracellular Ca^2+^-handling. To study this hypothesis, we developed a model of dissected intact muscle fibers obtained from human intercostal muscle biopsies and compared the intracellular Ca^2+^-handling of myotubes with that in adult muscle fibers. In addition to functional Ca^2+^-handling properties, we studied differences in gene expression, protein quantity and localization of Ca^2+^-handling proteins, and ultrastructural differences between the adult muscle fibers and myotubes. We show that viable, intact single muscle fibers can be obtained from human intercostal muscle biopsies and that these fibers display strictly regulated [Ca^2+^]_i_ transients and force production upon electrical stimulation ex vivo. In contrast, myotubes display only a rudimentary development of Ca^2+^-handling properties and do not contract. Consistent with this, we identify functionally important differences in the quantity and localization of Ca^2+^-handling proteins and ultrastructure between human muscle fibers and myotubes.

## Methods

### Study participants

Six recreationally active individuals suffering from lung cancer, four males and two females (age 65–76 years), scheduled for lobectomy at the Karolinska University Hospital in Stockholm, Sweden, were recruited for experiments on intercostal muscles. For patient characteristics see Additional file 1: Table S1. For the experiments on myoblasts and myotubes from vastus lateralis muscle biopsies, eight healthy recreationally active individuals, four males and four females (age 21–27 years), were recruited. The planned experiments and procedures were explained before subjects gave their written informed consent to participate. The study was approved by the Regional Ethical Review Board in Stockholm for experiments on intercostal muscles (Dnr 2012/2181-31/2) and for the experiments on myoblasts and myotubes (Dnr 2012/173-31/3).

### Muscle biopsies

External and internal intercostal muscle biopsies were obtained during thoracotomies. Biopsies were sampled at the midaxillary line in the fourth intercostal space during thoracoscopy or in the fifth intercostal space during open surgical procedures. Biopsies were collected with intact periostium at both ends to ensure non-disrupted muscle fibers and preserved tendons at both ends. Biopsies were placed in Dulbecco’s modified eagle medium (DMEM) (Gibco® Invitrogen, Life Technologies, Carlsbad, CA, USA) containing 0.2 % fetal bovine serum (FBS) (Gibco® Invitrogen, Life Technologies) bubbled with a mixture of 95 % O_2_ and 5 % CO_2_ prior to collection and immediately transported to the laboratory. Vastus lateralis muscle biopsies were obtained by the Bergström percutaneous needle biopsy technique [23].

### Muscle fiber dissection and mounting

Human internal and external intercostal muscle fibers are 0.5–1 cm long with diameters ranging from 30 to 100 μm. Intact single muscle fibers with intact tendons were dissected as previously described for mouse toe muscle fibers [24]. The isolated intercostal muscle fiber was mounted in a stimulation chamber between an Akers 801 force transducer and an adjustable holder, and the length was adjusted to that giving maximal tetanic force. The fiber was superfused with Tyrode solution (mM): NaCl, 121; KCl, 5.0; CaCl_2_, 1.8; MgCl_2_, 0.5; NaH_2_PO_4_, 0.4; NaHCO_3_, 24.0; EDTA, 0.1; glucose, 5.5. FBS (0.2 %) was added to the solution to improve cell survival. The solution was bubbled with a mixture of 5 % CO_2_ and 95 % O_2_, which gives an extracellular pH of 7.4. Experiments were performed at room temperature (approximately 24 **°**C).

### Muscle fiber force and [Ca^2+^]_i_ measurements

In muscle fibers, [Ca^2+^]_i_ was measured with the fluorescent Ca^2+^ indicator indo-1 (Indo 1, Invitrogen/Molecular Probes, Life Technologies), which was microinjected into the fiber. After injection of indo-1, the fiber was allowed to rest for at least 30 min. Tetanic force was then tested in the injected fibers and at regular intervals throughout the rest of the experiment, and if tetanic force declined by 10 % or more, the data from that fiber were discarded. Indo-1 was excited with light at 360 (±5) nm, and the light emitted at 405 (±5) and 495 (±5) nm was measured with two photomultiplier tubes. The fluorescence ratio of the light emitted at 405 nm to that emitted at 495 nm (R) is monotonically related to [Ca^2+^]_i_ according to the following equation (Eq. 1) [25]:

$$ {\left[{\mathrm{Ca}}^{2+}\right]}_{\mathrm{i}}={\mathrm{K}}_{\mathrm{D}}\upbeta \left(\mathrm{R}\hbox{--} {\mathrm{R}}_{\min}\right){\left({\mathrm{R}}_{\max }-\mathrm{R}\right)}^{-1} $$

where K_D_ is the apparent dissociation constant of indo-1, β is the ratio of the 495 nm signals at very low and saturating [Ca^2+^]_i_, and R_min_ and R_max_ are the ratios at very low and saturating [Ca^2+^]_i_, respectively. Tetanic force was measured as the mean over 100 ms where force was maximal.

### Isolation of myoblasts and cell culture

Human myoblasts were extracted from fresh muscle as previously described [2], with some modifications. Immediately following the biopsy procedure, approximately 100 mg of muscle tissue was placed in sterile phosphate buffered saline (PBS) (Gibco® Invitrogen, Life Technologies) containing 1 % antibiotic-antimycotic (ABAM) (Gibco® Invitrogen, Life Technologies) and incubated at 4 °C overnight. The following day, the muscle biopsy was incubated in 5 ml TrypLE™ Express Enzyme (1X) (Gibco® Invitrogen, Life Technologies) at 37 °C, 5 % CO_2_ with gentle agitation for 20 min. Undigested tissue was allowed to settle for 5 min at room temperature, and the supernatant containing the satellite cells were collected in 5 ml DMEM-F12 Glutamax (Gibco® Invitrogen, Life Technologies), containing 20 % FBS and 1 % ABAM. Digestion of the slurry was repeated twice. Subsequently, the supernatant containing the satellite cell population was collected. Isolated human myoblasts were cultured in DMEM-F12 Glutamax containing 20 % FBS and 1 % ABAM at 37 °C, 5 % CO_2_. Culture dishes were coated with collagen I (Collagen I, Bovine 5 mg/mL, Gibco® Invitrogen, Life Technologies) diluted to a final concentration of 50 μg/mL in 0.02 M acetic acid according to the manufacturer’s manual. Myoblasts were taken through serial passages to increase cell numbers prior to experimentation. All myoblasts were used for experimentation at passage 3–4. For experimentation, myoblasts were harvested and transferred to collagen I coated 35 mm glass-bottomed petri dishes (P35G-0-14-C, MatTek, Ashland, MA, USA) at a density of 8 × 10^4^ cells per dish. Myoblasts were allowed to settle for 24 h after which half of the dishes with plated myoblasts were used for intracellular Ca^2+^ measurements while the other half was differentiated into myotubes. Myotube differentiation was promoted by substituting the proliferation media with DMEM-F12 Glutamax containing 2 % FBS, 25 pM Insulin (I9278, Sigma-Aldrich, St. Louis, MO, USA) and 1 % ABAM.

### Immunomagnetic cell sorting

Enrichment of the cell population for myogenic cells was accomplished by a combination of pre-plating with magnetic activated cell sorting (MACS) separation as has previously been reported to produce a high yield of myogenic cells [26]. MACS separation of myogenic and non-myogenic cells was carried out as previously described for human muscle derived cells [27], with some modifications. Briefly, muscle derived cells plated in T75 flasks were incubated with primary antibody for CD56 (MY31, BD Biosciences, Franklin Lakes, NJ, USA) dissolved in DMEM-F12 Glutamax for 30 min at 37 °C, 5 % CO_2_. Cells were subsequently pelleted and re-suspended in PBS containing 0.1 % FBS and microbeads (Miltenyi Biotech, Lund, Sweden). Cell suspension was incubated in the dark at 4 **°**C for 15 min before being rinsed with PBS containing 0.1 % FBS and re-pelleted. Cells were magnetically separated using a midiMACS magnet and LS column (Miltenyi Biotech). The cells that were bound to the anti-CD56 microbeads complex were maintained in the column and constituted the positive (myogenic) fraction of cells. This fraction was subsequently plated and used for experimentation.

### Validation of myogenic origin of human myoblasts and myotube differentiation

At the time of plating cells for experimentation, a fraction of myoblasts was collected for confirmation of myogenic origin. Cells were spun down onto a cover glass for subsequent immunofluorescent staining of the myogenic marker desmin (D33, DAKO, Glostrup, Denmark). The fraction of desmin positive cells in the cell population was analyzed by dividing with the total number of nuclei stained with 4',6-diamidino-2-phenylindole dihydrochloride (DAPI) (Invitrogen/Molecular Probes) within each field. In the current study, 94 % (±1.32) of sub-confluent myoblasts were positive for desmin. To confirm myotube differentiation, cells were visually inspected and the presence of multinucleated (>2 nuclei) elongated cells positive for desmin was verified (Additional file 2: Figure S1A). Differentiation was further confirmed by an increase (*P* = 0.007) in mRNA levels of myogenin, a myogenic transcription factor involved in the terminal differentiation of myotubes [28] (Additional file 2: Figure S1B).

### Myoblast and myotube [Ca^2+^]_i_ measurements

In human myoblasts and myotubes, [Ca^2+^]_i_ was measured with the non-ratiometric fluorescent Ca^2+^ indicator fluo-3, which was loaded into cells in the acetoxymethyl ester form (Fluo-3 AM, Invitrogen, Life Technologies) and confocal microscopy using a modified Bio-Rad MRC 1024 unit attached to a Nikon Diaphot inverted microscope with a Nikon Plan Apo 20X objective (NA 1.3). Stored confocal images were analyzed with ImageJ (National Institutes of Health, http://rsb.info.nih.gov/ij). To enable comparisons between cells, changes in the fluo-3 fluorescence signal (ΔF) were divided by the fluorescence immediately before a stimulation pulse was given (F_0_).

### Cell stimulation protocol

Electrical stimulation was achieved by supramaximal current pulses (duration 0.5 ms) delivered from a lab built electrical stimulator via a pair of platinum plate electrodes lying parallel to the muscle fibers or along the edge of the glass bottom of the petri dish facing each other for myoblasts/myotubes. For chemical stimulation, myoblasts and myotubes were superfused for 1 min with Tyrode solution containing 5 mM adenosine triphosphate (ATP) (Sigma-Aldrich) or 1 mM 4-chloro-*m*-cresol (4-CmC) (Sigma-Aldrich). ATP- and 4-CmC-elicited changes in ΔF/F_0_ in myoblasts and myotubes were evaluated by comparing with changes in ΔF/F_0_ during exposure to normal Tyrode solution for the same time period.

### Quantification of the Ca^2+^ decay time constant

The Ca^2+^ decay time constant was analyzed by fitting a single exponential function plus a constant to the [Ca^2+^]_i_ transients of human myotubes and intercostal fibers stimulated with a single 70 Hz, 350 ms train of current pulses. For intercostal muscle fibers, original fluorescent ratios were smoothed one to two times using a seven-point quadratic polynomial to reduce noise prior to calculating [Ca^2+^]_i_ by Eq. 1. The peak Δ[Ca^2+^]_i_/[Ca^2+^]_i0_ for intercostal fibers or ΔF/F_0_ for myotubes, respectively, after the end of the stimulation period was set as the first point of the fit interval. The fit was continued until baseline (defined as <5 % of peak Δ[Ca^2+^]_i_/[Ca^2+^]_i0_ or ΔF/F_0_) was reached.

### Gene expression analysis

Total RNA was prepared by the Trizol method (Invitrogen, Life Technologies) and quantified spectrophotometrically by absorbance at 260 nm. One μg of total RNA from each sample was used for reverse transcription into cDNA for a final volume of 20 μl (High Capacity Reverse Transcription Kit, Applied Biosystems, Life Technologies). Real-time PCR (ABI-PRISMA 7700 Sequence Detector, Perkin-Elmer, Applied Biosystems, Life Technologies) procedures were employed to determine mRNA expression. Probes and primers (TaqMan) for SERCA1 (Hs01092295_m1), SERCA2 (Hs00544877_m1), RyR1 (Hs00166991_m1), α1s-DHPR (Hs00163885_m1), myogenin (Hs01072232_m1), MYH7 (Hs01110632_m1), MYH2 (HsHs0040042_m1), MYH1 (Hs00428600_m1), UBB (Hs00430290_m1), UBC (Hs00824723_m1), RPS18 (Hs01375212_g1), GAPDH (4352934E), 18S (4310893E), CTSB (Hs00157194_m1), and HK2 (Hs00606086_m1) were purchased from Applied Biosystems, Life Technologies. Reaction and amplification mixes (10 μl) consisted of the diluted (1:50) cDNA (4.5 μl), TaqMan Fast Universal PCR Master Mix (5.0 μl), and specific primers (0.5 μl). Subsequent cycling protocols were 2 min at 50 °C and 10 min at 90 °C followed by 40 cycles at 95 °C for 15 s and 60 °C for 1 min. To identify the optimal reference gene for normalization, the stability of the following reference genes was analyzed: UBB, UBC, RPS18, GAPDH, 18S, CTSB, and HK2. The geometrical mean of RPS18 and UBB was identified as the most stable reference according to the NormFinder algorithm [29], and target gene expression was therefore reported as a ratio to the geometrical mean of these two by the 2^−ΔCT^ formula.

### Protein extraction and Western blot

Cells and muscle bundles from intercostal muscle biopsies were homogenized in lysis buffer of the following composition (mM): 20 HEPES (pH 7.6), 150 NaCl, 5 EDTA, 1 Na_3_VO_4_ and 25 KF, 5 % glycerol (*v*/*v*), 0.5 % Triton X-100 (*v*/*v*), and protease inhibitor cocktail (one tablet/50 mL; Roche Diagnostics GmbH, Mannheim, Germany). Protein concentrations were subsequently determined using the Bradford technique. 50–60 μg of protein for myoblasts and myotubes or 15 μg per sample for intercostal muscles were loaded on 4–20 % SDS precast gels (Bio-Rad, Hercules, CA, USA) and separated through electrophoresis together with a protein ladder. Gels were transferred to PVDF-membranes using the Trans-Blot Turbo Transfer System from Bio-Rad. Blocking was completed using fluorescent blocking buffer (Merck Millipore, Darmstadt, Germany) during 60 min at room temperature (RT). Membranes were incubated overnight at 4 °C with primary antibodies (1:1000) for SERCA1 (ab2819, Abcam, Cambridge, FL, USA), SERCA2 (ab2861, Abcam), RyR (ab2868, Abcam) and/or (1:2000) for α1s-DHPR (sc-8160, Santa Cruz Biotechnology, Dallas, TX, USA). After the overnight incubation, membranes were washed (3 × 10 min) in PBST (0.1 %) and incubated with IRDye secondary antibody (LI-COR Biosciences, Cambridge, UK) for 60 min at RT. A final series of washes were then performed before scanning the membranes (Odyssey SA Infrared Imaging System, LI-COR Bioscience). The blots were subsequently quantified using ImageJ. Protein quantity was expressed as a ratio to total GAPDH abundance (1:2000; ab9485, Abcam).

### Immunofluorescent staining

Muscle fiber bundles containing four to five fibers were dissected manually and mounted in 35-mm petri dishes (P35G-0-14-C, MatTek). Myotubes and muscle fibers were fixed in 4 % formaldehyde for 20 min and then permeabilized by 0.3 % Triton X-100 in PBS solution. After rinsing, fibers were pre-incubated for 30 min in 4 % bovine serum albumin (BSA) (Sigma-Aldrich). Myotubes and muscle fibers were incubated overnight at 4 °C with primary antibodies (1:50) for SERCA1 (ab2819, Abcam), SERCA2 (ab2861, Abcam), RyR (MA3-925 (34C), Thermo Scientific, Waltham, MA, USA), α1s-DHPR (sc-8160, Santa Cruz) (dilution in PBS containing 1 % BSA and 0.1 % Triton-X) at 4 °C overnight. After the overnight incubation, myotubes and muscle fibers were washed (3 × 10 min) in PBS and incubated with secondary antibody (1:500) anti-mouse Alexa Fluor 568 and/or anti-goat Alexa Fluor 488 (Invitrogen, Life Technologies). Images of longitudinal thin sections of stained cells were obtained with laser confocal microscopy using a modified Bio-Rad MRC 1024 unit attached to a Nikon Diaphot inverted microscope with a Nikon Plan Apo 60X oil objective (NA 0.75). Excitation was at 491 and 561 nm, and the emitted light was collected through 522- and 605-nm narrowband filters. To provide an estimate of the overlap between RyR and α1s-DHPR signals in myotubes and intercostal fibers, the intensity plot of RyR and α1s-DHPR labeling was measured in ImageJ.

### Transmission electron microscopy

Human myotubes and intercostal muscle fibers were fixed in 2.5 % glutaraldehyde in 0.1 M phosphate buffer, pH 7.4. After fixation, cells were rinsed in 0.1 M phosphate buffer and centrifuged. The pellets were then post-fixed in 2 % osmium tetroxide in 0.1 M phosphate buffer, pH 7.4 at 4 °C for 2 h, dehydrated in ethanol followed by acetone and embedded in LX-112 (Ladd, Burlington, VT, USA). Ultrathin sections (approximately 50–60 nm) were cut by a Leica EM UC 6 (Leica, Vienna, Austria). Sections were contrasted with uranyl acetate followed by lead citrate and examined in a Tecnai 12 Spirit Bio TWIN transmission electron microscope (FEI Company, Eindhoven, Netherlands) 100 kV. Digital images were taken by using a Veleta camera (Olympus Soft Imaging Solutions, GmbH, Münster, Germany).

### Statistical analysis

SigmaPlot version 13.0 (Systat Software, Inc., San Jose, CA, USA) was used for statistical analysis. All variables were examined for normal distribution and were log- or square root-transformed before analysis as needed to better approximate a normal distribution. For comparisons between two data groups, two-tailed Student’s *t* test or Mann-Whitney Rank Sum test were used as appropriate. Statistically significant differences when comparing more than two groups were evaluated by the use of a one-way ANOVA. Tukey’s post hoc test was used to locate differences in mean values. Differences were considered significant at *P* < 0.05. Data are represented as means (± SEM) unless stated otherwise.

## Results

### Human muscle fibers and myotubes show marked differences in [Ca^2+^]_i_ transient kinetics and ability to contract

In intact muscle fibers, electrically evoked action potentials activate the voltage sensitive Ca^2+^ channels (DHPR) in the t-tubules that in turn triggers the opening of the sarcoplasmic reticulum (SR) Ca^2+^ release channel (RyR1), resulting in an increase in [Ca^2+^]_i_ and force production. In this way, the events following α-motoneuron activity in vivo can be recapitulated ex vivo [30]. We tested whether electrically stimulated human myotubes and myoblasts would also respond with an increase in [Ca^2+^]_i_ and compared the resultant [Ca^2+^]_i_ transients to those in human muscle fibers.

**Fig. 1:**
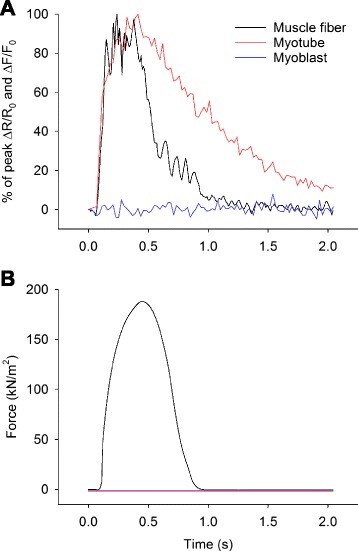
Muscle fibers and myotubes show marked differences in [Ca^2+^]_i_ transient kinetics and ability to contract. **a** Typical example of changes in indo-1 (muscle fiber) and fluo-3 signal (myotube and myoblast) displayed as percentage of maximum in response to a single 70 Hz, 350 ms train of current pulses for a human muscle fiber (*black*), myotube (*red*), and myoblast (*blue*, myoblast response shown as percentage of maximal myotube response). **b** Typical example of force production following a single 70 Hz, 350 ms train of current pulses in a human intercostal muscle fiber (*black*). A *flat line* representing myotube (*red*) and myoblast (*blue*) force production was added for illustrative purposes as none of these cells contracted in response to electrical stimulation

**Fig. 2:**
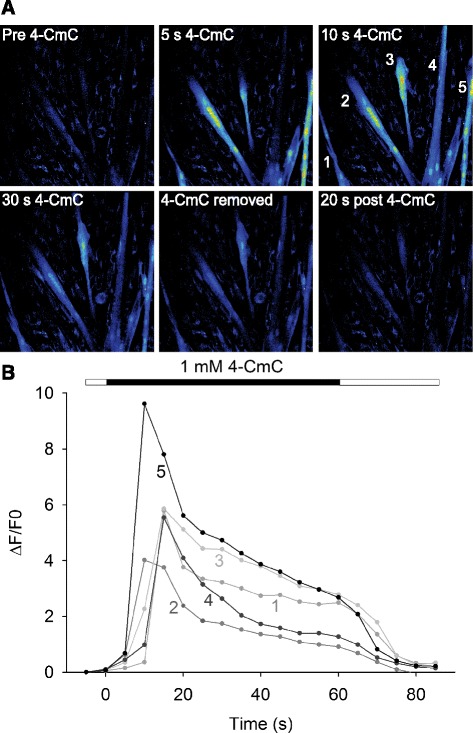
Pharmacological activation of the RyR markedly increases [Ca^2+^]_i_ but does not cause contractions in myotubes. **a**, **b** Typical example of changes in fluorescent signal induced by selective activation of the RyR1 by 4-CmC in myotubes; note the lack of contraction despite robust [Ca^2+^]_i_ increase and lack of [Ca^2+^]_i_ increase in surrounding mononuclear cells

Intact single muscle fibers were dissected from human intercostal muscle biopsies obtained during thoracotomies. Additionally, human myoblasts were extracted from biopsies obtained from the vastus lateralis muscle and differentiated to myotubes for 8 days. Cells were stimulated electrically with a single 70 Hz, 350 ms train of current pulses. Upon stimulation, the dissected single muscle fibers all responded with a transient increase in [Ca^2+^]_i_ and force production (Fig. 1a, b). The resultant increase in [Ca^2+^]_i_ and force production was reproducible over time (Additional file 3: Figure S2A, B). Mean basal [Ca^2+^]_i_ in human muscle fibers was 31 nM (±13) and mean plateau [Ca^2+^]_i_ was 1.0 μM (±0.2). Mean tetanic force per cross-sectional area generated was 405 kN/m^2^ (±112). While about half of the myotubes responded to electrical stimulation with a transient increase in fluo-3 signal (reflecting change in [Ca^2+^]_i_), none of the myoblasts showed any change in fluo-3 signal upon electrical stimulation (Fig. 1a). Interestingly, despite the marked increases in [Ca^2+^]_i_ upon electrical stimulation, no contraction of any myotube was observed. This was further investigated in myotubes exposed to 4-CmC which acts on the RyR to facilitate the release of Ca^2+^ from the SR [31] in order to fully activate SR Ca^2+^ release, and again, no contraction was observed even when [Ca^2+^]_i_ was maximally increased (Fig. 2a, b).

As the [Ca^2+^]_i_ transient duration has been demonstrated to be of importance for the downstream effects of Ca^2+^ [22, 21], the Ca^2+^-handling properties of human muscle fibers and myotubes were further investigated regarding the [Ca^2+^]_i_ transient kinetics. Aligned [Ca^2+^]_i_ transients demonstrated similar Ca^2+^ release kinetics between human muscle fibers and myotubes (Fig. 1a). However, the decay of the Ca^2+^ transients was markedly slower in human myotubes than in the muscle fibers (Fig. 1a). By fitting a single exponential function plus a constant to the [Ca^2+^]_i_ transient decay phase, the mean Ca^2+^ decay time constants were calculated. The mean Ca^2+^ decay time constant was approximately six times greater (888 ms (±66) vs. 146 ms (±13), *P* = 0.002) in myotubes than in muscle fibers.

### The abundance of Ca^2+^-handling proteins differs markedly between human muscle fibers, myoblasts, and myotubes

Differences in the relative abundance of Ca^2+^-handling proteins will affect the functional Ca^2+^-handling properties of muscle cells [32]. Thus, we hypothesized that the differences in functional Ca^2+^-handling observed between human muscle fibers, myoblasts, and myotubes would be explained by differences in the quantity of Ca^2+^-handling proteins. The mRNA and protein levels of RyR, DHPR, and the SR Ca^2+^ ATPases SERCA1 and SERCA2 were therefore analyzed in these cells.

**Fig. 3:**
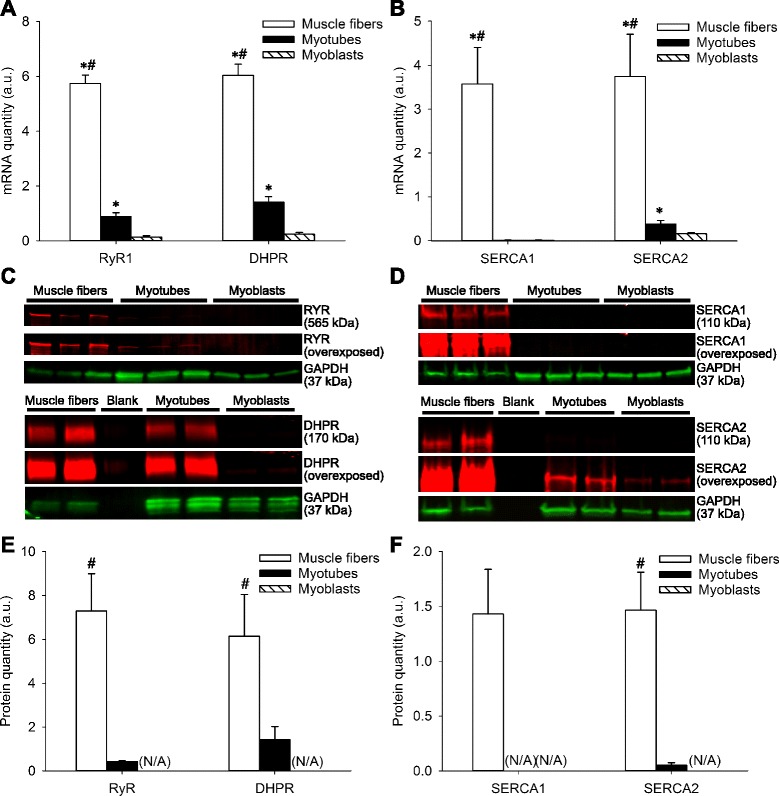
The abundance of Ca^2+^-handling proteins differs markedly between human muscle fibers, myotubes and myoblasts. mRNA quantities of **a** RyR1 and DHPR and **b** SERCA1 and SERCA2 in human muscle fibers myotubes and myoblasts. Representative Western blot bands for **c** RyR and DHPR and **d** SERCA1 and SERCA2. Densitometry for the protein quantity of **e** RyR and DHPR and **f** SERCA1 and SERCA2 in human muscle fibers myotubes and myoblasts. Data are presented as the mean ± SEM. *Asterisk* denotes *P* < 0.05 relative to myoblasts, and *number sign* denotes *P* < 0.05 relative to myotubes. (*N/A*) not applicable due to protein quantity to low for reliable measurements

The mRNA expression of RyR1 and DHPR was higher (*P* < 0.001) in muscle fibers than in either myotubes or myoblasts, and both were higher (*P* < 0.001) in myotubes than in myoblasts (Fig. 3a). In muscle fibers, the mRNA quantity of both SERCA1 and SERCA2 was markedly higher (*P* < 0.001) than in myotubes and myoblasts. SERCA2 mRNA was higher (*P* = 0.032) in myotubes than in myoblasts. SERCA1 mRNA abundance was very low in both myoblasts and myotubes; hence, it did not change (*P* = 0.869) as myoblasts differentiated into myotubes (Fig. 3b).

In muscle fibers, the protein quantity of RyR when normalized to GAPDH was approximately 17-fold higher (*P* < 0.001) and that of DHPR approximately fourfold higher (*P* = 0.039) than in myotubes (Fig. 3e). RyR protein was non-detectable in myoblasts, and DHPR only weakly detected in myoblasts upon deliberate overexposure of the membrane (typical example in Fig. 3c). While readily detected in muscle fibers, SERCA1 protein was non-detectable by Western blot in either myotubes or myoblasts even as the membrane was deliberately overexposed (typical example in Fig. 3d). The protein quantity of SERCA2 when normalized to GAPDH was approximately 28-fold higher in muscle fibers (*P* = 0.003) than in myotubes (Fig. 3f) and only weakly detected in myoblasts upon deliberate overexposure of the membrane (typical example in Fig. 3d).

Finally, we investigated the mRNA abundance of the slow type skeletal muscle/β-cardiac myosin heavy chain (MHC I), type IIa MHC (MHC IIa), and type IIx MHC (MHC IIx) in human muscle fibers, myotubes, and myoblasts. In muscle fibers, the mRNA quantity of MHC I (*P* = 0.004 and *P* < 0.001, respectively), MHC IIa (*P* < 0.001) and MHC IIx (*P* < 0.001) was higher than in both myotubes and myoblasts (Additional file 4: Figure S3A). Following differentiation of myoblasts into myotubes, there was an increase (*P* < 0.001) in the mRNA quantity of all MHC isoforms investigated from very low levels of expression in myoblasts (Additional file 4: Figure S3A). In both muscle fibers and myotubes, MHC IIa was the predominantly expressed MHC isoform at the mRNA level (Additional file 4: Figure S3B).

### The localization of Ca^2+^-handling proteins and ultrastructure differ markedly between human muscle fibers and myotubes

Myoblast differentiation into myotubes resulted in increased levels of Ca^2+^-handling proteins (Fig. 3a–d). One further step in the maturation process is the alignment of proteins into the arrangement required for fully functional Ca^2+^-handling in adult muscle fibers. To elucidate potential differences between human muscle fibers and myotubes in this regard, we used immunofluorescence staining and confocal microscopy to compare the localization of Ca^2+^-handling proteins. Additionally, transmission electron microscopy (TEM) was used to identify ultrastructural differences between human muscle fibers and myotubes.

**Fig. 4:**
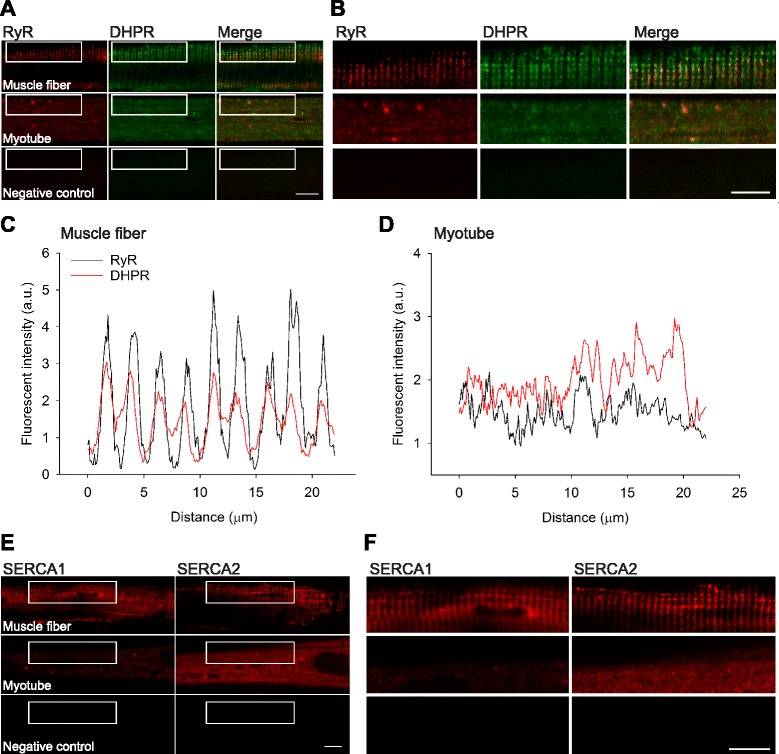
The localization of Ca^2+^-handling proteins differs markedly between human muscle fibers and myotubes. **a** Representative immunofluorescent staining for RyR and DHPR in human muscle fibers (*top row*) and myotubes (*middle row*). **b** Magnification of the area indicated in **a. c**, **d** Fluorescent intensity (a.u.) for RyR (*black*) and DHPR (*red*) in a human muscle fiber (*left*) and myotube (*right*). **e** Representative immunofluorescent staining for SERCA1 and SERCA2 in human muscle fibers (*top row*) and myotubes (*middle row*). **f** Magnification of the area indicated in **e**. *Scale bars* indicate 10 μm. Primary antibody was omitted for the respective negative controls (*bottom row* in **a**, **b** and **e**, **f**)

The protein localization of RyR and DHPR as well as SERCA1 and SERCA2 demonstrated a regular cross-striated pattern in human muscle fibers that was absent in myotubes (Fig. 4a–f). Double immunofluorescent staining for RyR and DHPR showed that there was less overlap of RyR and DHPR in human myotubes compared with muscle fibers as indicated by the intensity plot for RyR and DHPR (Fig. 4c, d). For SERCA1 and SERCA2, the signal intensity for SERCA1 was weaker than that for SERCA2 in myotubes (Fig. 4e, f), which is consistent with the Western blot results (Fig. 3d).

**Fig. 5:**
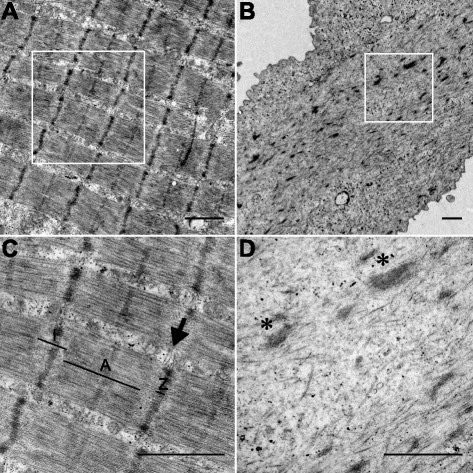
The ultrastructure differs markedly between human muscle fibers and myotubes. Representative TEM image of the ultrastructure in human intercostal muscle fibers (**a**, **c**) and human myotubes (**b**, **d**). **b** Note presence of areas containing rudimentary myofilament structures along with areas that lack even rudimentary myofilament structures in myotubes. **c**, **d** Magnification of the areas indicated in **a**, **b**, respectively. In human intercostal muscle fibers (**c**), the characteristic arrangement of myofibrils separated by triads (*arrow*) at the junction of the A (*A*) and I bands (*I*) that are absent in human myotubes (**d**), where only rudimentary myofilament structures (*asterisk*) can be seen. *Scale bars* indicate 1 μm

TEM images of human muscle fibers showed a highly organized regular repeating arrangement of myosin and actin filaments arranged in sarcomeres. The characteristic arrangement of myofibrils separated by the transverse tubule and terminal cisternae at either side that form triads at the junction of the A and I bands was clearly seen (Fig. 5a). In myotubes, myosin and actin filaments were sparse and did not display any regular arrangement. Areas of rudimentary myofilaments could be visualized together with extensive areas with little evidence of myofilament organization. Additionally, in myotubes there was little evidence of the fundamental unit of excitation-contraction coupling, transverse tubules adjoining terminal cisternae (Fig. 5b).

### Human myoblast differentiation into myotubes is paralleled by functional changes in intracellular Ca^2+^-handling

Myoblast differentiation into myotubes is associated with changes towards attaining morphological, metabolic, and biochemical properties similar to those in adult skeletal muscle [3, 4]. In addition, human myotubes but not myoblasts, respond to electrical stimulation by an increase in [Ca^2+^]_i_ (Fig. 1a), and myoblast differentiation into myotubes results in increased levels of Ca^2+^-handling proteins (Fig. 3a–d). As this indicates that differences in terms of intracellular Ca^2+^-handling exist between myoblasts and myotubes, we compared the functional Ca^2+^-handling properties of these cells.

**Fig. 6:**
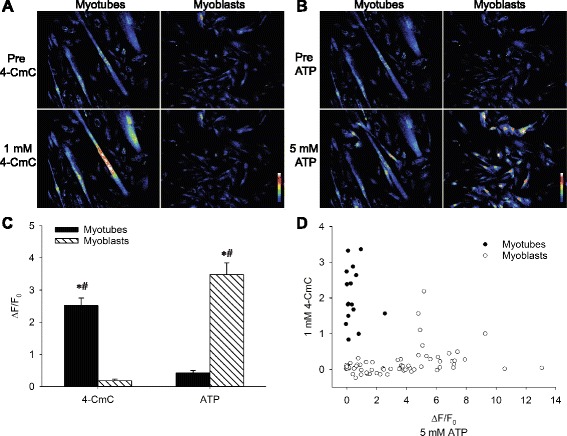
Human myoblast differentiation into myotubes is paralleled by functional changes in intracellular Ca^2+^-handling. **a** Typical example of human myotube and myoblast response to the selective RyR activator 4-CmC and **b** to the purinergic receptor agonist ATP. *Calibration bars* indicate scale of fluorescent intensities. **c** Maximal change in fluorescence expressed as ∆F/F_0_ following exposure of human myotubes and myoblasts to 4-CmC and ATP, respectively. Data are presented as the mean ± SEM. *Asterisk* denotes *P* < 0.05 compared with normal Tyrode throughout, and *number sign* denotes *P* < 0.05 relative to myoblasts or myotubes, respectively. **d** Maximal changes in fluorescent signal expressed as ΔF/F_0_ in response to 4-CmC vs. ATP in myotubes (*closed circles*) and myoblasts (*open circles*)

Myoblasts and myotubes were exposed to 4-CmC, which acts on the RyR to facilitate the release of Ca^2+^ from the SR [31]. In myotubes, stimulation with 4-CmC resulted in a robust increase (*P* < 0.001) in fluo-3 signal (Fig. 6a, c). In contrast, myoblasts demonstrated less of an increase in fluo-3 signal when exposed to 4-CmC and this increase did not reach statistical significance (*P* = 0.158) (Fig. 6a, c). Cells were also exposed to ATP, which acts via purinergic receptors to induce influx of extracellular Ca^2+^ into immature muscle cells [33]. ATP stimulation did not alter fluo-3 signal in myotubes (*P* = 0.917) but evoked a marked increase (*P* < 0.001) in fluo-3 signal in myoblasts (Fig. 6b, c). Figure 6d plots the response of each cell that was exposed to both ATP and 4-CmC and shows a clear pattern in that a marked response to one compound was associated with a minimal response to the other.

## Discussion

In the current study, we demonstrate the first measurements of [Ca^2+^]_i_ and force production in intact single human muscle fibers. This novel model was employed to compare the Ca^2+^-handling properties of human muscle fibers with that of an existing ex vivo model of human muscle constituted by human myotubes. The major novel findings of the current study are the following: (1) intact single muscle fibers that allow for continuous measurements of [Ca^2+^]_i_ and force production can be obtained from human intercostal muscle biopsies, (2) marked differences in intracellular Ca^2+^-handling exist between human muscle fibers and myotubes, and (3) these result from differences in the quantity and localization of Ca^2+^-handling proteins.

The basal and tetanic [Ca^2+^]_i_ together with specific forces reported here in human muscle fibers are similar to values reported previously in intact single rodent muscle fibers [24, 34]. However, the specific forces generated by the intact single human muscle fibers are somewhat larger than what generally has been reported for skinned human muscle fibers [35–38]. As skinned intercostal muscle fibers generate forces similar to those reported in skinned muscle fibers obtained from other muscle groups [36], disparities between muscle groups are unlikely to explain this difference. Instead, this is likely explained by other factors that distinguish the two preparations, such as the loss of soluble proteins or altered spacing of myosin and actin filaments associated with the skinning procedure [39]. This discrepancy underlines the importance of studying basic properties of human muscle in intact viable muscle fibers in addition to previously established models. Dissection of single human muscle fibers is technically challenging and requires human muscle biopsies with tendons intact at both ends. The current model is therefore unlikely to replace existing methods of clinical screening for diseased Ca^2+^-handling, such as malignant hyperthermia, but will provide a powerful tool for studies of Ca^2+^-dependent processes in human adult muscle. Moreover, the current model illustrates that mechanistic studies of the sort previously limited to animal models can be implemented also for studies of human skeletal muscle.

In adult skeletal muscle fibers, there is strictly controlled action potential-induced activation of the DHPR that, in turn, opens up the RyR1 to release Ca^2+^ from the SR [40, 41]. In the present study, approximately half of the investigated myotubes responded to electrical stimulation with an increase in [Ca^2+^]_i_. This is consistent with previous studies in human myotubes reporting depolarization-induced increases in [Ca^2+^]_i_ in approximately one third [15] to one half of myotubes examined [16]. These findings, together with the low protein expression and poor co-localization of DHPR and RyR found in the current study and by others [15, 17], show that the Ca^2+^-handling is functionally immature in human myotubes. Furthermore, the electron microscope images reveal that the ultrastructure, including the association between t-tubules and SR, is completely different in human muscle fibers and myotubes.

Further evidence of an immature Ca^2+^-handling in myotubes is the markedly slower decay of [Ca^2+^]_i_ transients in myotubes than in adult muscle fibers. This finding is in accordance with previous reports of a slow restoration of [Ca^2+^]_i_ to resting levels in immortalized human myotubes following depolarization [17]. These authors suggested that this was due to low levels of SERCA1 expression and our results confirm that there is little SERCA1 and SERCA2 at either the mRNA or protein level in myotubes. In addition, we demonstrate that the structured organization of SERCA1 and SERCA2 in muscle fibers is absent in myotubes. Collectively, the differences in protein quantity and localization of SERCA1 and SERCA2 between muscle fibers and myotubes provide an explanation to the observed difference in [Ca^2+^]_i_ transient decay. In addition, these differences may help explain the lower stimulation frequencies necessary to attain a fused tetanus in contracting human myotubes in comparison with muscle fibers [12–14]. Importantly, as diverse durations of [Ca^2+^]_i_ transients differentially affect the cellular signaling initiated by increased [Ca^2+^]_i_ [21, 22, 42–45], distinct rates of [Ca^2+^]_i_ transient decay in myotubes and muscle fibers, as demonstrated in the current study, will result in divergent effects on Ca^2+^ signaling pathways. Thus, when studying Ca^2+^-dependent processes such as gene expression and metabolism, myotubes are likely to provide different answers to those given by intact adult muscle fibers.

In the present myotubes, contractions were not observed when [Ca^2+^]_i_ was increased either by current pulses or 4-CmC application. This was explained by a low expression of sarcomeric MHCs and a lack of sarcomeric structures in human myotubes when analyzed by electron microscopy. The inability of human myotubes to contract is in accordance with the majority of previous studies and indicates a difference to rodent myotubes, which may contract when grown in culture [15–17]. Alternative culture protocols have attempted to grow myotubes that contract, including co-cultures with fetal rat spinal cord explants or neural agrin [15, 16], implementation of continuous electrical pulse stimulations into the culture protocol [5, 46], or bioengineering techniques [12]. These approaches have been partially successful in growing contracting human myotubes ex vivo [5, 12, 13, 46–48] and highlight the difficulty of growing human myotubes with characteristics similar to those of muscle fibers. Importantly, even though contracting human myotubes may be obtained in culture, they produce less contractile force, attain fused tetanus at lower stimulation frequencies, and exhibit kinetic parameters that are slower than those in muscle fibers [12–14].

While the Ca^2+^-handling is less mature in human myotubes than in muscle fibers, our results also demonstrate substantial differences regarding Ca^2+^-handling properties between human myoblasts and myotubes. Changes in responsiveness to ATP and 4-CmC as well as an induced expression of Ca^2+^-handling proteins were observed as myoblasts were differentiated into myotubes. The purinergic receptor agonist ATP has previously been demonstrated to increase [Ca^2+^]_i_ in human myoblasts and in the early process of myotube differentiation, with a reduced responsiveness to ATP in later developmental stages of myotube differentiation [33]. Conversely, 4-CmC has been demonstrated to be a specific activator of the SR Ca^2+^ release channel RyR1 [31] and would be expected to increase [Ca^2+^]_i_ in myogenic cells with a functional SR. The current findings of increased [Ca^2+^]_i_ upon stimulation with 4-CmC in myotubes therefore indicates a maturation of intracellular Ca^2+^-handling towards that in adult muscle fibers. Moreover, the present results suggest that myoblasts and myotubes are functionally distinct regarding their Ca^2+^-handling properties and can reliably be distinguished by their divergent responses to ATP and 4-CmC. This is in line with previous reports of other properties acquired following differentiation of myoblasts into myotubes that are similar to those in adult muscle [3, 4].

## Conclusions

Intact single muscle fibers that display strictly regulated [Ca^2+^]_i_ transients and force production upon electrical stimulation can be obtained by careful dissection of human intercostal muscle biopsies. In contrast, despite a more mature Ca^2+^-handling in myotubes than in myoblasts, human myotubes lack important aspects of adult Ca^2+^-handling. We conclude that human myotubes are an inappropriate model for human adult muscle when studying Ca^2+^-dependent processes, such as gene expression and metabolic processes. These results provide insights to some important functional differences between adult muscle fibers and myotubes and suggest caution when translating results obtained in human myotubes to muscle fibers.

## Additional files

Additional file 1: Table S1. Patient characteristics. Additional file 2: Figure S1. Confirmation of myotube formation. (**A**) Typical example of desmin staining (red) and DAPI (blue) showing myotube formation at 8 days of differentiation. Scale bars indicate 200 μm. (**B**) mRNA quantity of myogenin, a myogenic transcription factor involved in the terminal differentiation of myotubes, in myoblasts (dashed bar) and myotubes (black bar). Data are presented as the mean ± SEM. Asterisk denotes *P* < 0.05. Additional file 3: Figure S2. Confirmation of reproducibility of [Ca^2+^]_i_ transients and force production in human muscle fibers. Typical example of reproducibility of [Ca^2+^]_i_ transients and force production following electrical stimulation in a human intercostal muscle fiber. (**A**) Percentage of peak ΔR/R_0_ (representing [Ca^2+^]_i_) and (**B**) force production in a human intercostal muscle fiber following a single 70 Hz 350 ms train of current pulses. Blue lines represent recordings following the first tetanus 30 min after Indo-1 injection, and red lines represent recordings 8 min thereafter. Additional file 4: Figure S3. mRNA expression of slow type skeletal muscle/β-cardiac, type IIa and type IIx MHCs in human muscle fibers, myotubes and myoblasts. The mRNA quantity of MHC I, MHC IIa, and MHC IIx differs markedly between human muscle fibers, myotubes and myoblasts. (**A**) mRNA quantities of MHC I, MHC IIa, and MHC IIx in human muscle fibers, myotubes, and myoblasts. Data are arbitrary units where the values have been adjusted so that the mRNA quantities of the different MHC isoforms are approximately equal in muscle fibers so to better visualize the differences within each MHC isoform between the different cell types. Data are presented as the mean ± SEM. Aterisk denotes *P* < 0.05 relative to myoblasts, and number sign denotes *P* < 0.05 relative to myotubes. (**B**) mRNA quantities of the different MHC isoforms in human muscle fibers and myotubes. Data are presented as the mean ± SEM. Aterisk denotes *P* < 0.05 relative to MHC IIx, and number sign denotes *P* < 0.05 relative to MHC IIa.

## Abbreviations

[Ca^2+^]_i_: cytosolic free [Ca^2+^]
18S: 18S ribosomal RNA
4-CmC: 4-chloro-*m*-cresol
ABAM: antibiotic-antimycotic
ATP: adenosine triphosphate
BSA: bovine serum albumin
CTSB: cathepsin B
DAPI: 4',6-diamidino-2-phenylindole dihydrochloride
DHPR: dihydropyridine receptor
DMEM: Dulbecco’s modified eagle medium
FBS: fetal bovine serum
GAPDH: glyceraldehyde-3-phosphate dehydrogenase
HK2: hexokinase 2
MACS: magnetic activated cell sorting
MHC I: slow type skeletal muscle/β-cardiac myosin heavy chain
MHC IIa: type IIa myosin heavy chain
MHC IIx: type IIx myosin heavy chain
MYH: myosin heavy chain
PBS: phosphate buffered saline
RPS18: ribosomal protein S18
RyR: ryanodine receptor
SERCA: sarcoplasmic/endoplasmic reticulum Ca^2+^ ATPase
SR: sarcoplasmic reticulum
TEM: transmission electron microscopy
UBB: ubiquitin B
UBC: ubiquitin C
