# Supplementary material for: Intracellular Ca2+-handling differs markedly between intact human muscle fibers and myotubes
Source: Skelet Muscle. 2015 Aug 20;5:26. doi: 10.1186/s13395-015-0050-x (PMC4545874; doi:10.1186/s13395-015-0050-x)
Supplement: Additional file 1: Table S1. — Patient characteristics. [file 13395_2015_50_MOESM1_ESM.pdf]

Table S1. Patient characteristics.

Patient #	Medications	Comorbidities
**1**	Enalapril, Amlodipine	Hypertension, smoking
**2**	--	Prostate cancer
**3**	Oxycodone, Paracetamol, Ethylmorphine,	--
**4**	Furix, Metoprolol, Amlodipine, Oxycodone, Paracetamol, Oxazepam	Hypertension
**5**	Bisoprolol, Enalapril, Simvastatin, Trombyl	Atrial fibrillation, nephrosclerosis, previous CABG, smoking
**6**	Movicol, Trombyl, Metoprolol, Amlodipine, Losartan, Nitrofurantoin, Oxycodone, Gabapentine, Ipratropium bromide/salbutamol	Previous SAH, previous spinal infarction

Abbreviations: CABG (coronary artery bypass graft), SAH (subarachnoid hemorrhage).
