# Supplementary figures and images for: Intracellular Ca2+-handling differs markedly between intact human muscle fibers and myotubes
Source: Skelet Muscle. 2015 Aug 20;5:26. doi: 10.1186/s13395-015-0050-x (PMC4545874; doi:10.1186/s13395-015-0050-x)

**A**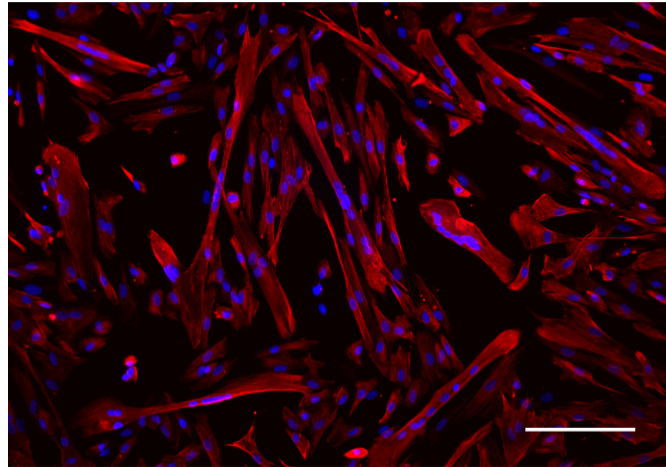**B**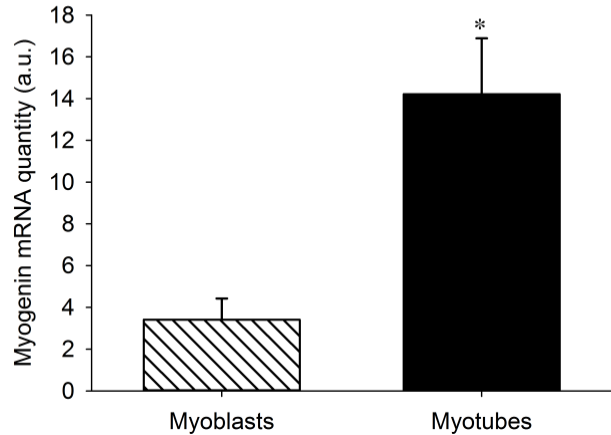

Supplement: Additional file 2: Figure S1. — Confirmation of myotube formation. (A) Typical example of desmin staining (red) and DAPI (blue) showing myotube formation at 8 days of differentiation. Scale bars indicate 200 μm. (B) mRNA quantity of myogenin, a myogenic transcription factor involved in the terminal differentiation of myotubes, in myoblasts (dashed bar) and myotubes (black bar). Data are presented as the mean ± SEM. Asterisk denotes P < 0.05. [file 13395_2015_50_MOESM2_ESM.pdf]

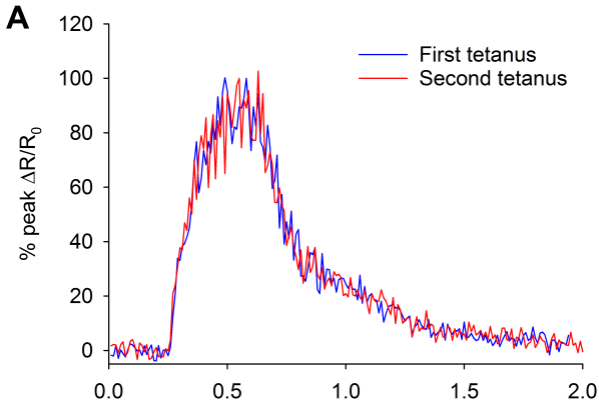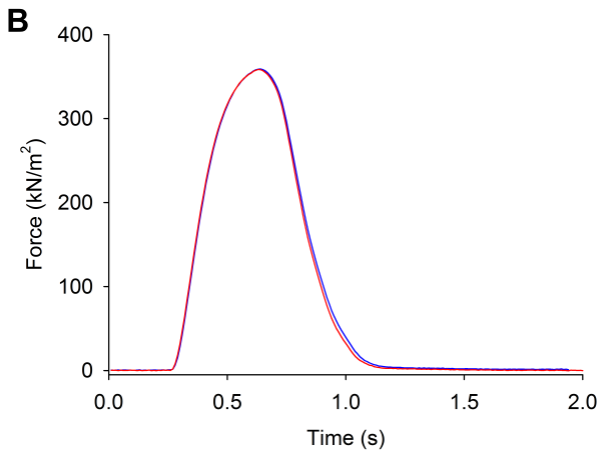

Supplement: Additional file 3: Figure S2. — Confirmation of reproducibility of [Ca2+]i transients and force production in human muscle fibers. Typical example of reproducibility of [Ca2+]i transients and force production following electrical stimulation in a human intercostal muscle fiber. (A) Percentage of peak ΔR/R0 (representing [Ca2+]i) and (B) force production in a human intercostal muscle fiber following a single 70 Hz 350 ms train of current pulses. Blue lines represent recordings following the first tetanus 30 min after Indo-1 injection, and red lines represent recordings 8 min thereafter. [file 13395_2015_50_MOESM3_ESM.pdf]

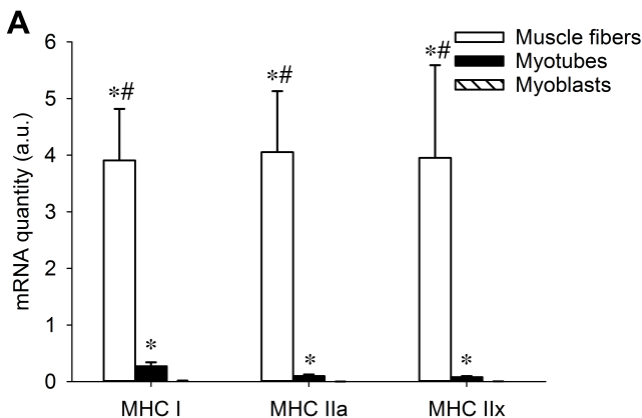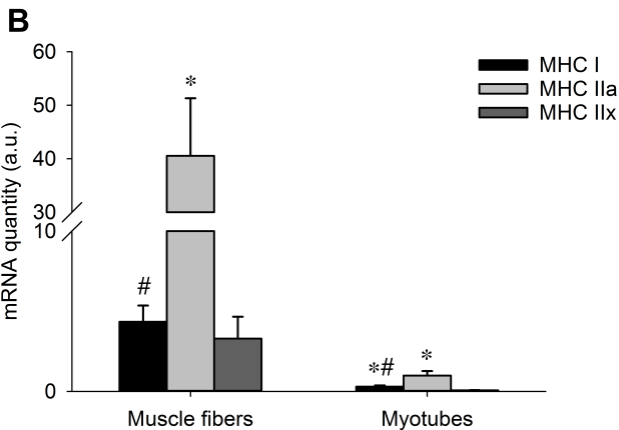

Supplement: Additional file 4: Figure S3. — mRNA expression of slow type skeletal muscle/β-cardiac, type IIa and type IIx MHCs in human muscle fibers, myotubes and myoblasts. The mRNA quantity of MHC I, MHC IIa, and MHC IIx differs markedly between human muscle fibers, myotubes and myoblasts. (A) mRNA quantities of MHC I, MHC IIa, and MHC IIx in human muscle fibers, myotubes, and myoblasts. Data are arbitrary units where the values have been adjusted so that the mRNA quantities of the different MHC isoforms are approximately equal in muscle fibers so to better visualize the differences within each MHC isoform between the different cell types. Data are presented as the mean ± SEM. Aterisk denotes P < 0.05 relative to myoblasts, and number sign denotes P < 0.05 relative to myotubes. (B) mRNA quantities of the different MHC isoforms in human muscle fibers and myotubes. Data are presented as the mean ± SEM. Aterisk denotes P < 0.05 relative to MHC IIx, and number sign denotes P < 0.05 relative to MHC IIa. [file 13395_2015_50_MOESM4_ESM.pdf]
